# Hypoglycemia-associated In-stent Thrombosis: Are We Doing Too Much?

**DOI:** 10.7759/cureus.1712

**Published:** 2017-09-25

**Authors:** Uyanga Batnyam, Nway Ko Ko, Aamir Javaid

**Affiliations:** 1 Internal Medicine Residency Program, University of Central Florida College of Medicine; 2 Internal Medicine, University of Central Florida College of Medicine

**Keywords:** in-stent thrombosis, diabetes, myocardial infaction, hypoglycemia

## Abstract

Diabetes mellitus is one of the most common modifiable risk factors for coronary artery disease, and its prevalence is rising globally. Persistent hyperglycemia has a well-established cardiovascular risk, and its treatment plays an important role in the prevention of future cardiovascular events. While we improved microvascular complications such as retinopathy, nephropathy, and neuropathy by stringent blood glucose control, the cardiovascular morbidity and mortality in diabetics remain high. Hypoglycemia, on the other hand, is an important side effect of pharmaceutical blood glucose control, especially those who are treated with insulin. Here, we report the case of a 38-year-old man with type 1 diabetes presenting twice with acute ST-elevation myocardial infarction, both in the setting of documented hypoglycemia. There are reported cases of acute cardiovascular events or silent myocardial ischemia associated with hypoglycemia, and we wish to raise awareness for clinicians who treat this special population of patients.

## Introduction

The prevalence of diabetes mellitus (DM) is continuously rising; approximately 30.3 million adults in the United States have been diagnosed with DM [1]. Persistent hyperglycemia is a known cardiovascular risk factor, and by tight blood glucose control, improvement in microvascular complications such as retinopathy, nephropathy, and neuropathy has been demonstrated. However, the cardiovascular morbidity and mortality in diabetics remain high despite tight blood glucose control. Hypoglycemia is not an uncommon presentation among patients presenting with acute coronary syndrome (ACS), and we are unaware if that is the cause or the consequence. Multiple large studies have demonstrated poor cardiovascular outcomes with intensive blood glucose control, and there have been reported associations between hypoglycemia, myocardial ischemia, and ACS [2-3].

## Case presentation

**Figure 1:**
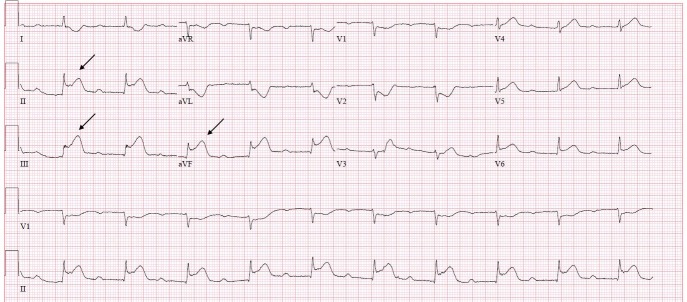
Electrocardiogram upon presentation ST elevation (see arrows) in inferior leads (II, III and aVF) with reciprocal changes.

**Figure 2:**
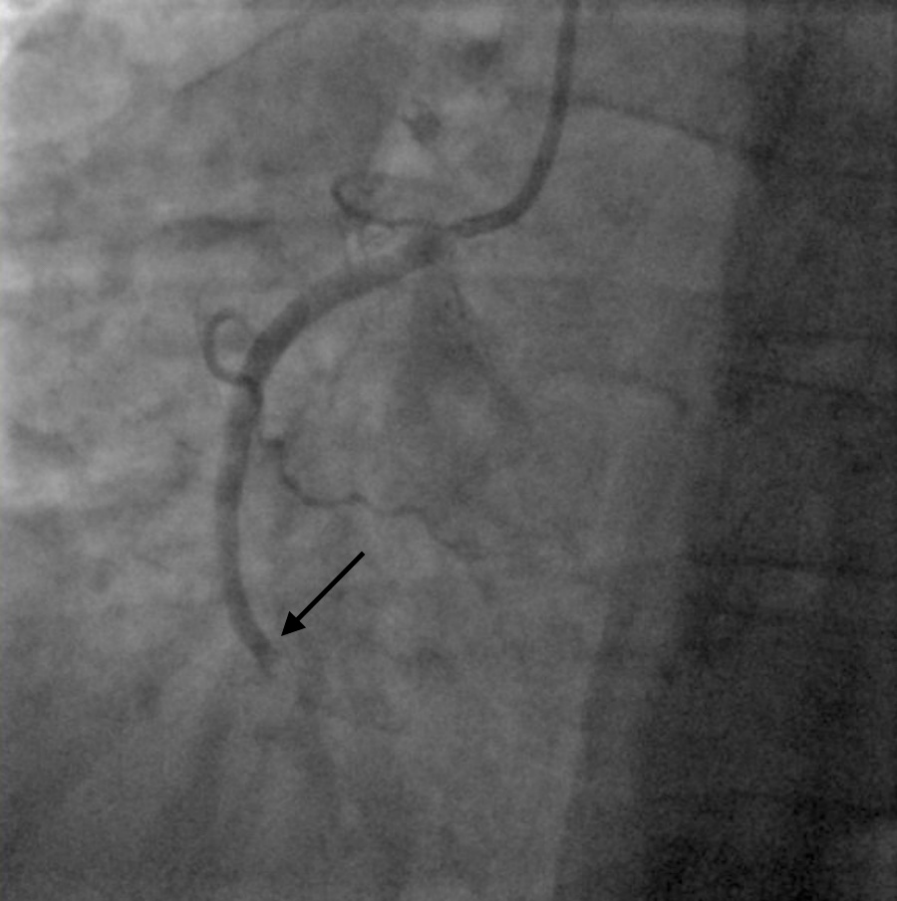
Total occlusion of right coronary artery An emergent left heart catheterization revealed total occlusion of the right coronary artery in the previously placed stent (arrow). There was no flow distal to the occlusion.

**Figure 3:**
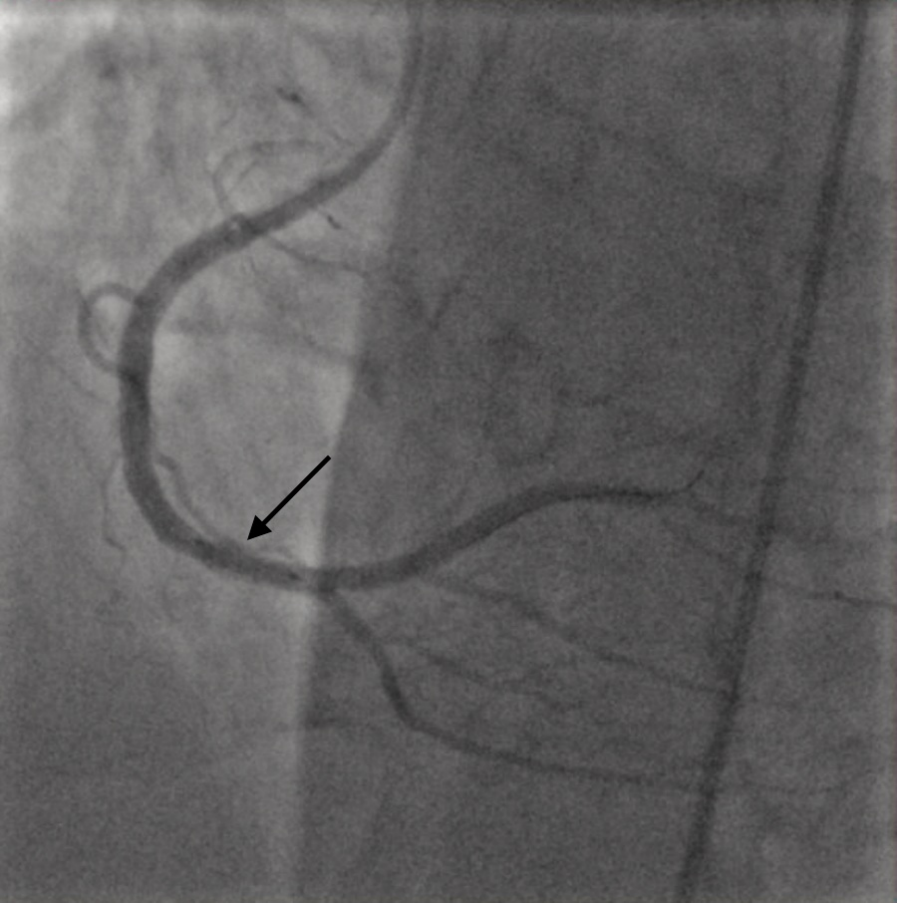
Right coronary artery, post-stent placement Right coronary artery with TIMI-3 flow (arrow) after mechanical thrombus aspiration and placement of another stent distal to the previously placed stent.

A 38-year-old male with type 1 diabetes mellitus presented to our institute with severe substernal chest pain that started shortly after regaining consciousness from a short duration of hypoglycemic coma with documented blood glucose of 40 mg/dL. The patient had active chest pain upon presentation, and the electrocardiogram showed ST segment (isoelectric section of the electrocardiogram between the end of the S wave and the beginning of the T wave) elevations in the inferior leads with reciprocal changes (Figure 1). An emergent left heart catheterization revealed an in-stent thrombosis in his previously placed nine-month-old stent despite being strictly compliant with a dual antiplatelet therapy including aspirin and ticagrelor (Figure 2). Of interest, his first ST-elevation myocardial infarction nine months ago also was preceded by a hypoglycemic episode with documented low blood sugar levels, and he subsequently had a drug-eluting stent (DES) implanted in the right coronary artery. We had performed mechanical thrombus aspiration, and placed another DES distal to the previous stent (Figure 3). 

## Discussion

The relationship between hypoglycemia and poor cardiovascular outcomes is complex. Hypoglycemia activates the sympathoadrenal system, causes glucagon and catecholamine surges, thereby causing increased cardiac work and myocardial oxygen demand. Hypoglycemia has been shown to induce inflammatory cascades, and has been associated with enhanced platelet aggregation which is known to promote thrombosis [4-5]. Case reports and early animal models have hypothesized the association between hypoglycemia and ACS, and multiple studies have revealed a documented association between silent myocardial ischemia on a cardiac monitor during asymptomatic hypoglycemic episodes that is recorded by a continuous glucose monitoring in diabetics [6]. There are multiple case reports of ACS in the setting of acute insulin poisoning, particularly in younger adults without established cardiovascular risk factors [7]. Hypoglycemia induced by oral hypoglycemic agents have also been reported to be associated with ACS [8-9]. It is possible that in some cases ACS can be the consequence of hypoglycemia, and the “dead in bed Syndrome” in diabetics could be associated with hypoglycemia-induced adverse cardiovascular events. Physicians might be doing more harm by intense blood glucose control, and large studies are needed to evaluate this potential association. 

## Conclusions

To the best of our knowledge, we report the first case of hypoglycemia associated stent thrombosis in a patient with type 1 diabetes mellitus. We are proposing special awareness for hypoglycemia in patients with diabetes who are medically treated.
